# Validity of the work productivity and activity impairment questionnaire - general health version in patients with rheumatoid arthritis

**DOI:** 10.1186/ar3141

**Published:** 2010-09-22

**Authors:** Wei Zhang, Nick Bansback, Annelies Boonen, Adam Young, Amitabh Singh, Aslam H Anis

**Affiliations:** 1 Centre for Health Evaluation and Outcome Sciences, St Paul's Hospital, 620-1081 Burrard Street, Vancouver, BC V6Z 1Y6, Canada; 2 School of Population and Public Health, University of British Columbia, 5804 Fairview Avenue, Vancouver, BC V6T 1Z3, Canada; 3 Department of Internal Medicine, Division of Rheumatology, University Hospital Maastricht and Caphri Research Institute, P Debyelaan 25, 6229 HX Maastricht, The Netherlands; 4 Department of Rheumatology, St Albans City Hospital, Waverley Road, St Albans, Herts AL3 5PN, UK; 5 Inflammation Market Access, Pfizer, Inc. 500 Arcola Road, Dock E-4303 Collegeville, PA 19426, USA

## Abstract

**Introduction:**

The Work Productivity and Activity Impairment (WPAI) questionnaire is a well validated instrument to measure impairments in work and activities. However, its validation among patients with rheumatoid arthritis (RA) has not been well established. The present study's purpose is to evaluate the construct validity of the WPAI-general health version among RA patients and its ability to differentiate between RA patients with varying health status.

**Methods:**

Patients who were enrolled in the Early Rheumatoid Arthritis Network cohort and were employed at their most recent follow-up were recruited into this sub-study. A questionnaire battery incorporating the WPAI was administered along with a number of health outcomes including the Multidimensional Health Assessment Questionnaire, fatigue and patient assessment of disease activity. The construct validity of the WPAI was tested by the correlations between the WPAI and the health outcomes and other measures of productivity. Student's *t *tests were used to identify whether the WPAI outcomes differed between the two levels of heath status based on the median of health outcomes.

**Results:**

A total of 150 patients completed the WPAI questionnaire. The average age was 52 years old and the disease duration was 37.5 months since the first rheumatology visit. Of the 137 patients who were working for pay, 26 reported missing work in the past week due to their health problem, accounting for 45.5% of their working time (absenteeism). While 123 patients were working, 24% of their work was impaired due to their health problem (presenteeism). In addition, 33% of the patients' regular daily activities (activity impairment) had been prevented due to their health problems. There were moderate correlations between the WPAI absenteeism and function, pain, fatigue, and disease severity (r = 0.34 to 0.39). The WPAI presenteeism and activity impairment were strongly correlated with the health outcomes (0.67 to 0.77). Patients with more severe disease status (for example, low/high functional disability by median) had significantly higher absenteeism (4%/15%), presenteeism (15%/39%), and activity impairment (19%/53%) than those with less severe disease status.

**Conclusions:**

The WPAI is a valid questionnaire for assessing impairments in paid work and activities in RA patients and for measuring the relative differences between RA patients with different health status.

## Introduction

Rheumatoid arthritis (RA) is the most common form of inflammatory arthritis with a prevalence rate of about 1% and an annual incidence of 3 per 10,000 adults [1]. There is considerable evidence that RA can impact patients' productivity even during the very early phase of the disease. According to Burton *et al*., the time between RA onset until 50% probability of being permanently work disabled varied from 4.5 to 22 years [2]. Merkesdal *et al*. found that within the first three years of RA, there was an average of 82 days of sick leave per person-year and 26% of patients lost work because of RA [3]. Sick leave was more significant in the first year with an average of 113 days. In a study on patients with inflammatory joint conditions present for <12 months, Geuskens *et al*. found that 26% of all patients and 35% of the patients with RA reported more than two weeks of sick leave in the past six months [4]. In terms of the impact of RA on unpaid work, a recent clinical trial showed that at baseline patients with RA reported 9.2 missed days of household work and 11.2 days with productivity less than or equal to 50% in household work in one month [5]. Another trial at baseline found that RA patients got about 11 hours of unpaid or paid help to take over their unpaid work [6]. In the literature, functional disability has consistently been found to be associated with work disability [7-9]. In addition, pain and poor physical functioning were also associated with increased sick leave and reduced productivity at work [4,10].

In general, the impact of RA on paid work includes employed people missing time from work (absenteeism), reduced performance while at work (presenteeism), reduced routine working hours through changing or even losing jobs (employment status change). The impact on unpaid work usually refers to the impact of health problems on regular daily activities such as household work, shopping, and child care.

The Work Productivity and Activity Impairment (WPAI) questionnaire is an instrument to measure impairments in both paid work and unpaid work [11,12]. It measures absenteeism, presenteeism as well as the impairments in unpaid activity because of health problem during the past seven days. It has been validated to quantify work impairments for numerous diseases such as asthma, psoriasis, irritable bowel syndrome (IBS), ankylosing spondylitis (AS) and Crohn's disease [12-15]. In addition, the WPAI questionnaire has been used to compare work impairments between treatment groups in clinical (studies and) trials or between subjects with different disease severity levels [13-18]. However, the validation of this instrument among patients with RA has not been well established.

The objective of the study is to evaluate the construct validity of the WPAI-general health version (WPAI-GH) among RA patients and its ability to differentiate between RA patients with varying health status.

## Materials and methods

### Study design

This study is a cross-sectional study. Patients were recruited from a UK based registry of RA, the Early Rheumatoid Arthritis Network (ERAN), which is a group of rheumatology centres in the UK and Eire with an interest in treatment patterns and outcome in patients with recently diagnosed RA in normal clinical settings. Patients had already consented to take part in the ERAN research study. Patients who reported they were employed at their most recent follow-up with ERAN were invited to participate in this substudy. Those who agreed were sent a questionnaire battery. The questionnaire battery including the WAPI-GH and health outcome measures was administered at one time point. The WPAI-GH was used to measure the patients' work impairments. Ethical approval was gained from West Herts Multi-centre Research Ethics Committee, UK.

### WPAI-GH outcomes

The WPAI-GH consists of six questions: 1 = currently employed; 2 = hours missed due to health problems; 3 = hours missed other reasons; 4 = hours actually worked; 5 = degree health affected productivity while working (using a 0 to 10 Visual Analogue Scale (VAS)); 6 = degree health affected productivity in regular unpaid activities (VAS) [11,12]. The recall period for the questions 2 to 6 is seven days. Four main outcomes can be generated from the WPAI-GH and expressed in percentages by multiplying the following scores by 100: 1) percent work time missed due to health = Q2/(Q2 + Q4) for those who were currently employed; 2) percent impairment while working due to health = Q5/10 for those who were currently employed and actually worked in the past seven days; 3) percent overall work impairment due to health Q2/(Q2 + Q4) + ((1 - Q2/(Q2 + Q4)) × (Q5/10)) for those who were currently employed; 4) percent activity impairment due to health Q6/10 for all respondents [11,12]. For those who missed work and did not actually work in the past seven days, the percent overall work impairment due to health will be equal to the percent work time missed due to health.

### Construct validity: relation with health

We selected a number of instruments measuring health status that we believed would be correlated with productivity outcomes. The Multidimensional Health Assessment Questionnaire (MDHAQ) is a validated one-page questionnaire including a measure of functional disability, pain, and patient global health estimate [19]. The scoring of the MDHAQ was as follows: a) Function score: 10 activities of daily living (ADL) were scored 0 to 3, 0 = "without any difficulty", 1 = "with some difficulty", 2 = "with much difficulty", and 3 = "unable to do." To be consistent with the Health Assessment Questionnaire score (HAQ), the sum of 10 ADL scores was divided by 10 to give a score of 0 to 3; b) Pain VAS; c) Patient global estimate VAS on health impact. Fatigue VAS was used to measure patient assessment of fatigue problem. Patient global assessment (PtGA) of disease activity was used as a proxy of disease activity. Previous studies have found a strong correlation between the patient global assessment of disease activity and the disease activity score including 28-joint counts, with a VAS score greater than 40 indicating high disease activities [20]. All the VAS scales were presented as 21 circles to facilitate scoring without a ruler and an arithmetic scale of 0 to 10 in 0.5 unit increments was printed below the circles.

### Construct validity; relation with other measure of productivity

Questions on productivity adapted from alternative questionnaires were also included to assess the consistency of responses. They included the number of absent workdays in the past three months, a question adapted from the PROductivity and DISease Questionnaire (PRODISQ) [21], and questions adapted from Health and Labour Questionnaire (HLQ) [22,23] asking about lost hours due to presenteeism (the difference between the numbers of hours actually worked in the past seven days and the estimated number of hours used to complete the same work if patients did not experience any health problems) and the impact of health on unpaid work activities (hours of getting help with unpaid work activities in the past seven days).

In addition, the ability of the WPAI-GH to discriminate between better and worse health states was tested by dividing patients into two groups based on the median of the scores for function, pain, health impact, fatigue and disease activity (Better status: ≤median; Worse status: >median).

### Analysis

We measured the extent to which WPAI productivity outcomes were correlated with health status outcomes and the productivity questions adapted from alternative questionnaires. Due to the skewed nature of the productivity outcome data, nonparametric correlation (Spearman's correlation coefficient) was used to assess construct validity.

For a comparison between two groups, the effect size, the standardized mean difference between two groups on a measured outcome, was calculated for all WPAI-GH outcomes. An effect size of one indicates a change in magnitude equivalent to one standard deviation. According to Cohen [24,25], the absolute value of effect sizes (d) can be categorized as small (d = 0.2 to 0.5), medium (d = 0.5 to 0.8), or large (d > 0.8). A larger effect size indicates better discriminative ability. Student *t *tests were used to identify whether WPAI outcomes differed between the two levels of heath status. Wilcoxon tests were also used due to the skewed nature of the productivity outcome data.

## Results

A total of 354 patients believed to be in some sort of work in ERAN were contacted for the study and 186 (53%) agreed to take part in the study and were sent the questionnaires. One hundred and fifty-two patients sent back the questionnaire but two of these patients did not respond to the questions in the WPAI so that no WPAI outcomes could be calculated. Therefore, a total of 150 patients were included in the analysis. Among the 150 patients, the average age was 52 years old and 72% were female (Table 1). The sample's disease duration was 37.5 months since their first rheumatology visit. Patients had relatively mild function (0.6), pain (3.6), fatigue (4.6) and disease activity (3.6). Of the 137 (91%) patients that were working for pay, 26 (19%) reported missing work (absenteeism) in the past week due to their health, accounting for 45.5% of their working time (Table 2). While 123 patients were working, 24% of their actual work was impaired due to their health problem (presenteeism) with only 34 (28%) patients reported no such loss. In addition, 33% of the patients' regular daily activities had been prevented due to their health problems.

**Table 1:** Demographics and health status

Variables (*n *= 150)	Mean (SD)	Median (Q1 to Q3)
Age	52.1 (10.0)	52.9 (45.6 to 60.0)
Duration since onset of symptom (months)	48.7 (23.2)	46.0 (33.0 to 60.0)
Duration since first clinic visit (months)	37.5 (18.3)	35.7 (23.7 to 50.9)
Function (0 to 3)	0.6 (0.5)	0.5 (0.2 to 0.9)
Pain (0 to 10)	3.6 (2.5)	3.0 (1.5 to 5.5)
Pt global estimate (0 to 10)	3.0 (2.4)	2.5 (1.0 to 5.0)
Fatigue (0 to 10)	4.6 (2.9)	5.0 (2.0 to 7.0)
PtGA (0 to 10)	3.6 (2.6)	3.0 (1.5 to 5.5)
	***n***	**%**
Female	108	72.0

Pt global estimate, patient global estimate on health impact; PtGA, patient global assessment of disease activity; Q1, first quartile; Q3, third quartile

**Table 2:** WPAI outcomes

Variables	*n*	Mean (SD)	Median (Q1 to Q3)
*Patients working for pay*	*137*		
Percent work time missed due to health	136	8.7 (25.2)	0 (0 to 0)
Percent work time missed due to health (those with missed time >0)	26	45.5 (41.2)	18 (12 to 100)
*Patients who actually worked in the past seven days*	*123*		
Percent impairment while working due to health	122	24.0 (22.7)	20 (0 to 40)
Percent impairment while working due to health (those with % impairment while working >0)	88	33.3 (20.1)	30 (20 to 50)
Percent overall work impairment due to health	135	29.1 (29.8)	20 (0 to 46)
*All patients*	*150*		
Percent activity impairment due to health	150	33.3 (27.6)	30 (10 to 60)
Percent activity impairment due to health (those with % activity impairment >0)	123	40.7 (25.1)	30 (20 to 60)

Q1, first quartile; Q3, third quartile

For the correlation analysis between the WPAI productivity outcomes and the health status outcomes, all the correlations were in the logical direction and were highly significant (Table 3). There were moderate correlations between percent work time missed due to health and function, pain, fatigue, health impact, and disease severity (0.34 to 0.39). The other three WPAI outcomes were strongly correlated with all of the health status outcomes (0.67 to 0.77).

**Table 3:** Spearman correlations between WPAI outcomes and health status outcomes

	Function	Pain	Pt global estimate	Fatigue	PtGA
Percent work time missed	0.39	0.36	0.36	0.37	0.34
Percent impairment while working	0.69	0.75	0.74	0.67	0.76
Percent overall work impairment	0.67	0.73	0.71	0.68	0.73
Percent activity impairment	0.73	0.77	0.77	0.68	0.77

Pt global estimate, patient global estimate on health impact; PtGA, patient global assessment of disease activity

The number of absent workdays in the past three months was about 4.4 (standard deviation (SD): 10.5) days. The number of hours lost due to presenteeism in the past seven days was 1.4 (2.8) hours. Overall, patients got 4.0 (8.3) hours of help with their unpaid work activities in the past seven days. The percent of work time missed due to health was moderately correlated with number of absent workdays in the past three months (r = 0.56). The correlation between impairment while working and hours lost due to presenteeism was 0.39 and that between activity impairment and hours of getting help on unpaid work activities was 0.39.

When the patients were divided into two groups according to the median of each health status outcome variable, each of WPAI productivity outcomes was significantly lower among patients with better health status than patients with worse health status (Table 4). For example, patients with low functional disability (lower than median) had a significantly lower percentage of work time missed due to health (4 vs. 15), lower percentage of impairment while working due to health (15 vs. 39), and lower percentage of activity impairment due to health (19 vs. 53) than those with high functional disability. According to the effect size, WPAI outcomes had large discriminative abilities (1.10 to 1.79) except the percent work time missed due to health which showed a medium discriminative ability (0.43 to 0.63). The test results from *t *tests and Wilcoxon tests agreed (only the results of the *t* tests were reported).

**Table 4:** WPAI outcomes between two patient groups defined by the median for each health status outcome

		**Function**	**Pain**	**Pt global estimate**	**Fatigue**	**PtGA**
	
Percent work time missed	Better	4.2 (19.0)^‡^	1.5 (7.2)	2.0 (7.7)^†^	3.9 (15.8)^‡^	1.6 (7.1)
	Worse	15.5 (31.5)	16.3 (34.0)	15.6 (33.9)	14.5 (32.4)	16.9 (34.6)
	*Effect size*	*0.46*	*0.61*	*0.55*	*0.43*	*0.63*
Percent impairment while working	Better	14.5 (18.8)	11.6 (15.0)	10.9 (13.1)	12.8 (16.8)	11.7 (14.3)
	Worse	39.1 (20.1)	39.6 (21.1)	40.0 (21.7)	38.7 (21.0)	41.2 (21.0)
	*Effect size*	*1.27*	*1.56*	*1.66*	*1.38*	*1.69*
Percent overall work impairment	Better	17.6 (24.8)	12.2 (16.7)	11.8 (15.7)	14.8 (22.1)	12.3 (16.1)
	Worse	46.5 (28.2)	47.4 (30.0)	47.2 (30.3)	46.0 (28.9)	48.9 (30.1)
	*Effect size*	*1.10*	*1.46*	*1.48*	*1.22*	*1.55*
Percent activity impairment	Better	19.0 (21.1)	15.7 (17.0)	15.1 (17.0)	19.6 (21.0)	17.5 (19.0)
	Worse	52.7 (23.3)	51.9 (24.4)	52.0 (23.7)	49.9 (25.6)	51.9 (24.5)
	*Effect size*	*1.53*	*1.73*	*1.79*	*1.30*	*1.58*

Pt global estimate, patient global estimate on health impact; PtGA, patient global assessment of disease activity

If not indicated, *t *test *P *values <0.001; ^†^, *t *test *P *values <0.01; ^‡^, *t *test *P *values <0.05

Better status, ≤median; Worse status, >median. The median for each health status outcome was shown in Table 1

## Discussion

This is the first study to examine the construct validity of the WPAI-GH in a relatively large sample of patients with RA. The results support that the WPAI-GH displays construct validity as measured by moderate to strong correlations with all health status measures in terms of functional disability, pain, fatigue and disease activity (Table 3). As such, it appears that the WPAI productivity outcomes are assessing constructs that are relevant and important to patients with RA. However, the WPAI is moderately correlated with other productivity outcomes such as hours lost due to presenteeism and hours of getting help on unpaid work activities measured using the questions adapted from the HLQ. This may suggest that it measures another aspect of presenteeism and unpaid work productivity in comparison with the HLQ. In addition, according to the effect size analysis and paired test, the WPAI-GH could discriminate across health status with worse score being associated with measures indicating worse health status among the patients with RA. The results demonstrate the validity of the WPAI-GH is consistent with previous WPAI validation results for other diseases.

It is interesting that absenteeism (percent of work time missed) correlates less with health than presenteeism (impairment while working). This may be because the decision to stay home depends on more contextual factors than impairments while working that are related to the diseases; for example, the type of work, the fear to be a burden to colleagues, attitude towards work, consequence for income, fear to lose work, and so on. This suggests that absenteeism measures another dimension of the impact of the disease which is not measured by other health outcome instruments. One previous item response theory study in AS showed that work participation did not fit the unidimensionality of all other categories/domains of the International Classification of Functioning, Disability and Health that refer to body functions, body structures, activities and participation [26]. There is also the role of the social security system that is expected to influence more absenteeism than presenteeism/activity impairment, which makes absenteeism less comparable internationally [27]. However, the high correlation between impairment while working/activity impairment and other health outcomes may be partially attributable to the fact that they all use the same measuring 0 to 10 scale.

Among health outcomes, pain and PtGA were more correlated with work impairment and activity impairment than function and fatigue. In the literature, pain was found to be highly associated with reduced productivity at work [10]. However, no previous studies have shown that disease activity is highly correlated with work and activity impairment among people with RA. Further studies are needed to confirm the relationships.

There are two aspects of productivity that are important to measure: first, the work difficulties or work impairments due to health which influence work related quality of life and/or psychosocial impacts such as job satisfaction and stress, and second, the actual productivity losses due to health. The WPAI was designed predominantly to measure the first aspect but has been used in the past to measure and value the productivity losses for economic evaluations. However, the WPAI does not necessarily capture sufficient information to comprehensively measure actual productivity losses. To do so, it requires measures of actual lost time estimators as well as contextual factors such as job type, workplace team dynamics and compensation mechanism (a manuscript from Zhang *et al. *- in submission).

A total of 186 patients agreed to participate in the study but 150 patients completed WPAI questions and were included in our analysis. To identify whether there were non-response biases, we compared patient characteristics, including age, gender, duration since onset of symptom, duration since fist clinic visit, as well as disease activity score, HAQ and PtGA measured in the most recent follow-up with ERAN, between the 150 patients who were included in the analysis and the 36 patients who were not. No significant differences were found.

A limitation of this study is that no independent employment measure (gold standard) of missed work hours or work impairment while working was used as a validation criterion. Severens *et al*. investigated the agreement between registered and reported sick leave [28]. They demonstrated that 95% of the reported days of sick leave matched registered data perfectly when the recall period was limited to two and four weeks. This percentage decreased to 87%, 57%, and 51% for 2, 6, and 12 months, respectively. Reilly *et al*. assessed the accuracy of self-reported work hours missed measured by the WPAI:IBS version using retrospective diary among patients with IBS [13]. The high convergence of the WPAI:IBS and retrospective diary suggested that self-reports of percentage work hours missed during the past week were accurate.

In terms of work impairment while at work, presenteeism, Lerner *et al*. established the relationship between their Work Limitation Questionnaire and objective work productivity measures in two types of occupations [29]. However, it is quite difficult to measure the relationship between self-reported and objective measures of productivity at work because the objective measures varied with occupations as well as workplaces and so is the relationship [30]. Zhang *et al*. have demonstrated that the work productivity loss while at work varies widely with the approach chosen among people with arthritis [31,32]. Future research should focus on assessing the relationship between self report and objective measures of productivity loss and examining which approach provides more accurate measure of productivity loss.

We did not test the reliability of the WPAI-GH among patients with RA in this study. The test-retest reliability of the WPAI has been well established in populations with different diseases [33,34]. We assumed that this would not be different for the population with RA. The responsiveness of the WPAI-GH among patients with RA will be tested in a future study.

This is not the first application of WPAI among RA patients. One application of WPAI in RA was a study published as an abstract at 2008 American College of Rheumatology annual scientific meeting [35]. It was used to explore the impact of health problems on presenteeism in patients recently diagnosed with RA as compared to a healthy comparison group. Another application of WPAI was conducted by the Canadian Arthritis Network work productivity study, which was to compare different presenteeism measures among people with arthritis [31,32]. However, in both studies, only the question measuring impairment while working (question 5 of WPAI) was used and the main purposes were not to validate the instrument.

The WPAI has been used to measure work productivity in other diseases such as asthma, psoriasis, irritable bowel syndrome (IBS), ankylosing spondylitis (AS) and Crohn's disease [13-18]. The mean percent work time missed has been reported to be 5.0 for people with asthma [18], 4.4 for people with IBS [13], 9.0 for people with AS [14] and 18.3 for people with Crohn's disease [15], respectively, compared with 8.7 for people with RA in our study population. The percent impairment while working has been reported to be 20.0 in asthma, 15.5 in psoriasis [17], 32.4 in IBS, 41.7 in AS and 40.5 in Crohn's disease compared with 24.0 in RA from our study. Correspondingly, percent activity impairment has been found to be 32.0, 23.7, 41.4, 54.9 and 52.0 compared with 33.3 in our study of RA patients. According to these WPAI outcomes, it shows that RA has a relatively moderate impact on work productivity compared to other diseases. However, even for a certain disease, the WPAI outcomes vary with different levels of severity. Given that our RA population has early disease and therefore typically mild disease severity, the other disease populations compared may have a relatively more severe disease status. Simple comparisons should be made with caution.

## Conclusions

Construct validity and the discriminative ability of the WPAI-GH have been established in this study. Thus, the WPAI-GH is a valid questionnaire for assessing impairments in paid work and activities in RA patients and for measuring the relative differences between RA patients with different health status. The WPAI-GH is useful for measuring productivity outcomes in clinical practice. Further studies are needed to validate a tool for valuing productivity loss in economic analyses.

## Abbreviations

ADL: activities of daily living; AS: ankylosing spondylitis; ERAN: Early Rheumatoid Arthritis Network; HLQ: Health and Labour Questionnaire; IBS: irritable bowel syndrome; MDHAQ: Multidimensional Health Assessment Questionnaire; PRODISQ: PROductivity and DISease Questionnaire; PtGA: Patient Global Assessment; RA: rheumatoid arthritis; VAS: Visual Analogue Scale; WPAI: Work Productivity and Activity Impairment questionnaire; WPAI-GH: WPAI-general health version.

## Competing interests

This study was funded by Wyeth (now Pfizer). The Arthritis Research Centre of Canada, where AHA is a senior research scientist, has received a research grant from Wyeth. ERAN has received funding from Wyeth Pharmaceuticals and the Healthcare Commission. AS was an employee of Wyeth and owned its stocks/shares at the time of the study.

## Authors' contributions

WZ, NB, AY and AHA were involved in the conceptualization, design, data acquisition and interpretation. AB and AS participated in the conceptualization and data interpretation. WZ performed data analysis and drafted the manuscript. NB, AB, AY, AS and AHA critically revised the manuscript. All authors read and approved the final manuscript.

## References

[B1] Gabriel SE The epidemiology of rheumatoid arthritis Rheum Dis Clin North Am 2001 27 269 281 10.1016/S0889-857X(05)70201-5 11396092

[B2] Burton W Morrison A Maclean R Ruderman E Systematic review of studies of productivity loss due to rheumatoid arthritis Occup Med (Lond) 2006 56 18 27 10.1093/occmed/kqi171 16286432

[B3] Merkesdal S Ruof J Schoffski O Bernitt K Zeidler H Mau W Indirect medical costs in early rheumatoid arthritis: composition of and changes in indirect costs within the first three years of disease Arthritis Rheum 2001 44 528 534 10.1002/1529-0131(200103)44:3<528::AID-ANR100>3.0.CO;2-U 11263766

[B4] Geuskens GA Hazes JM Barendregt PJ Burdorf A Work and sick leave among patients with early inflammatory joint conditions Arthritis Rheum 2008 59 1458 1466 10.1002/art.24104 18821663

[B5] Osterhaus JT Purcaru O Richard L Discriminant validity, responsiveness and reliability of the rheumatoid arthritis-specific Work Productivity Survey (WPS-RA) Arthritis Res Ther 2009 11 R73 10.1186/ar2702 19457255 PMC2714119

[B6] Zhang W Bansback N Guh D Li X Nosyk B Marra CA Anis AH Short-term influence of adalimumab on work productivity outcomes in patients with rheumatoid arthritis J Rheumatol 2008 35 1729 1736 18688916

[B7] de Croon EM Sluiter JK Nijssen TF Dijkmans BA Lankhorst GJ Frings-Dresen MH Predictive factors of work disability in rheumatoid arthritis: a systematic literature review Ann Rheum Dis 2004 63 1362 1367 10.1136/ard.2003.020115 15479885 PMC1754825

[B8] Eberhardt K Larsson BM Nived K Lindqvist E Work disability in rheumatoid arthritis--development over 15 years and evaluation of predictive factors over time J Rheumatol 2007 34 481 487 17299844

[B9] Kessler RC Maclean JR Petukhova M Sarawate CA Short L Li TT Stang PE The effects of rheumatoid arthritis on labor force participation, work performance, and healthcare costs in two workplace samples J Occup Environ Med 2008 50 88 98 10.1097/JOM.0b013e31815bc1aa 18188086

[B10] Geuskens GA Hazes JM Barendregt PJ Burdorf A Predictors of sick leave and reduced productivity at work among persons with early inflammatory joint conditions Scand J Work Environ Health 2008 34 420 429 19137203 10.5271/sjweh.1298

[B11] Reilly MC Zbrozek AS Dukes EM The validity and reproducibility of a work productivity and activity impairment instrument Pharmacoeconomics 1993 4 353 365 10.2165/00019053-199304050-00006 10146874

[B12] Reilly Associates Health Outcomes Research http://www.reillyassociates.net

[B13] Reilly MC Bracco A Ricci J Santoro J Stevens T The validity and accuracy of the Work Productivity and Activity Impairment questionnaire - Irritable bowel syndrome version (WPAI:IBS) Alimentary Pharmacology and Therapeutics 2004 20 459 467 10.1111/j.1365-2036.2004.02091.x 15298641

[B14] Reilly MC Gooch KL Wong RL Kupper H van der Heijde D Validity, reliability and responsiveness of the Work Productivity and Activity Impairment Questionnaire in ankylosing spondylitis Rheumatology (Oxford) 2010 49 812 819 10.1093/rheumatology/kep457 20100797

[B15] Reilly MC Gerlier L Brabant Y Brown M Validity, reliability, and responsiveness of the work productivity and activity impairment questionnaire in Crohn's disease Clin Ther 2008 30 393 404 10.1016/j.clinthera.2008.02.016 18343277

[B16] Revicki DA Willian MK Menter A Gordon KB Kimball AB Leonardi CL Langley RG Kimel M Okun M Impact of adalimumab treatment on patient-reported outcomes: results from a Phase III clinical trial in patients with moderate to severe plaque psoriasis J Dermatolog Treat 2007 18 341 350 10.1080/09546630701646172 18058494

[B17] Pearce DJ Singh S Balkrishnan R Kulkarni A Fleischer AB Feldman SR The negative impact of psoriasis on the workplace J Dermatolog Treat 2006 17 24 28 10.1080/09546630500482886 16467020

[B18] Chen H Blanc PD Hayden ML Bleecker ER Chawla A Lee JH TENOR Study Group Assessing productivity loss and activity impairment in severe or difficult-to-treat asthma Value Health 2008 11 231 239 10.1111/j.1524-4733.2007.00229.x 18380635

[B19] Pincus T Swearingen C Wolfe F Toward a multidimensional Health Assessment Questionnaire (MDHAQ): assessment of advanced activities of daily living and psychological status in the patient-friendly health assessment questionnaire format Arthritis Rheum 1999 42 2220 2230 10.1002/1529-0131(199910)42:10<2220::AID-ANR26>3.0.CO;2-5 10524697

[B20] Kobelt G Lindgren P Lindroth Y Jacobson L Eberhardt K Modelling the effect of function and disease activity on costs and quality of life in rheumatoid arthritis Rheumatology (Oxford) 2005 44 1169 1175 10.1093/rheumatology/keh703 15956093

[B21] Koopmanschap MA PRODISQ: a modular questionnaire on productivity and disease for economic evaluation studies Expert Rev Pharmacoecon Outcomes Res 2005 5 23 28 10.1586/14737167.5.1.23 19807557

[B22] van Roijen L Essink-Bot ML Koopmanschap MA Bonsel G Rutten FF Labor and health status in economic evaluation of health care. The Health and Labor Questionnaire Int J Technol Assess Health Care 1996 12 405 415 10.1017/S0266462300009764 8840661

[B23] The health and labour questionnaire (manual) http://publishing.eur.nl/ir/repub/asset/1313/bmgimt20000609160629.pdf

[B24] Cohen J A power primer Psychol Bull 1992 112 155 159 10.1037/0033-2909.112.1.155 19565683

[B25] Cohen J Statistical Power Analysis for the Behavioural Sciences 1988 2 Hillsdale, NJ: Lawrence Erlbaum Association

[B26] Cieza A Hilfiker R Boonen A van der Heijde D Braun J Stucki G Towards an ICF-based clinical measure of functioning in people with ankylosing spondylitis: a methodological exploration Disabil Rehabil 2009 31 528 537 10.1080/09638280802173475 18608418

[B27] Boonen A van der Heijde D Landewe R Spoorenberg A Schouten H Rutten-van Molken M Guillemin F Dougados M Mielants H de Vlam K van der Tempel H van der Linden S Work status and productivity costs due to ankylosing spondylitis: comparison of three European countries Ann Rheum Dis 2002 61 429 437 10.1136/ard.61.5.429 11959767 PMC1754093

[B28] Severens JL Mulder J Laheij L Verbeek V Precision and accuracy in measuring absence from work as a basis for calculating productivity costs in The Netherlands Social Science and Medicine 2000 51 243 249 10.1016/S0277-9536(99)00452-9 10832571

[B29] Lerner D Amick BC Lee JC Rooney T Rogers WH Chang H Berndt ER Relationship of employee-reported work limitations to work productivity Med Care 2003 41 649 659 10.1097/00005650-200305000-00012 12719689

[B30] Prasad M Wahlqvist P Shikiar R Shih YC A review of self-report instruments measuring health-related work productivity: a patient-reported outcomes perspective Pharmacoeconomics 2004 22 225 244 10.2165/00019053-200422040-00002 14974873

[B31] Zhang W Bansback N Beaton D Lacaille D Gignac M Badley E Bombardier C Anis AH How is reduced performance at work (presenteeism) associated with measures of disease severity in patients with osteoarthritis (OA) and rheumatoid arthritis (RA)? [abstract] Ann Rheum Dis 2008 67 583 10.1136/ard.2008.092015

[B32] Zhang W Gignac MA Beaton D Tang K Anis AH Canadian Arthritis Network Work Productivity Group Productivity loss due to presenteeism among patients with arthritis: estimates from 4 instruments J Rheumatol 2010 37 1805 1814 10.3899/jrheum.100123 20595270

[B33] Bushnell DM Reilly MC Galani C Martin ML Ricci JF Patrick DL McBurney CR Validation of electronic data capture of the Irritable Bowel Syndrome--Quality of Life Measure, the Work Productivity and Activity Impairment Questionnaire for Irritable Bowel Syndrome and the EuroQol Value Health 2006 9 98 105 10.1111/j.1524-4733.2006.00087.x 16626413

[B34] Ciconelli RM Soarez PC Kowalski CC Ferraz MB The Brazilian Portuguese version of the Work Productivity and Activity Impairment: General Health (WPAI-GH) Questionnaire Sao Paulo Med J 2006 124 325 332 10.1590/S1516-31802006000600005 17322953 PMC11068280

[B35] Braakman-Jansen LM Taal E Kuper I van de Laar MA Productivity loss at work in patients with early rheumatoid arthritis [abstract] American College of Rheumatology annual scientific meeting (presentation 1275) 2008

